# Adolescent emotional responses to different music arrangements

**DOI:** 10.3389/fpsyg.2025.1583665

**Published:** 2025-11-12

**Authors:** Yaming Wei, Huijuan Wu, Qianqi Fan

**Affiliations:** 1 Research Center for Arts and health, Xiamen Humanity Hospital, Xiamen, Fujian, China; 2 School of Humanities and Social Sciences, Fuzhou University, Fuzhou, Fujian, China; 3 Music (Music Therapy), Sookmyung Womans University, Seoul, Republic of Korea; 4 Henan Normal University, Xinxiang, Henan, China

**Keywords:** adolescent emotions, music arrangement, gender differences, music preference, positive and negative affect schedule, emotional response

## Abstract

**Objective:**

This study aims to investigate adolescents’ positive emotional responses to different music arrangements, focusing on the influence of gender, music preference, and personal musical experience on emotional responses. The hypothesis proposes that gender and musical experience have a significant impact on adolescents’ emotional responses to music.

**Methods:**

The study recruited 120 adolescents who listened to three different arrangements of the same song. Emotional responses were assessed using the Positive and Negative Affect Schedule (PANAS), alongside physiological measures including electrodermal activity (EDA) and heart rate variability (HRV). Statistical analyses, including analysis of variance (ANOVA) and correlation analysis, were employed to evaluate the effects of gender, music preference, and personal musical experience on emotional responses.

**Results:**

Female participants showed higher PANAS scores for certain music arrangements. Music education or artistic training had no significant effect. Music preference was negatively correlated with emotional responses, while listening experience had a significant positive impact. Physiologically, the rock version (B) elicited the highest EDA, the classical version (A) showed the highest HRV, and the bossa nova version (C) yielded moderate responses in both measures.

**Conclusion:**

Gender and personal music preferences play important roles in adolescents’ emotional responses to musical arrangements. These findings hold potential applications in music therapy, education, and psychological health interventions for adolescents.

## Graphical Abstract

**Figure:**
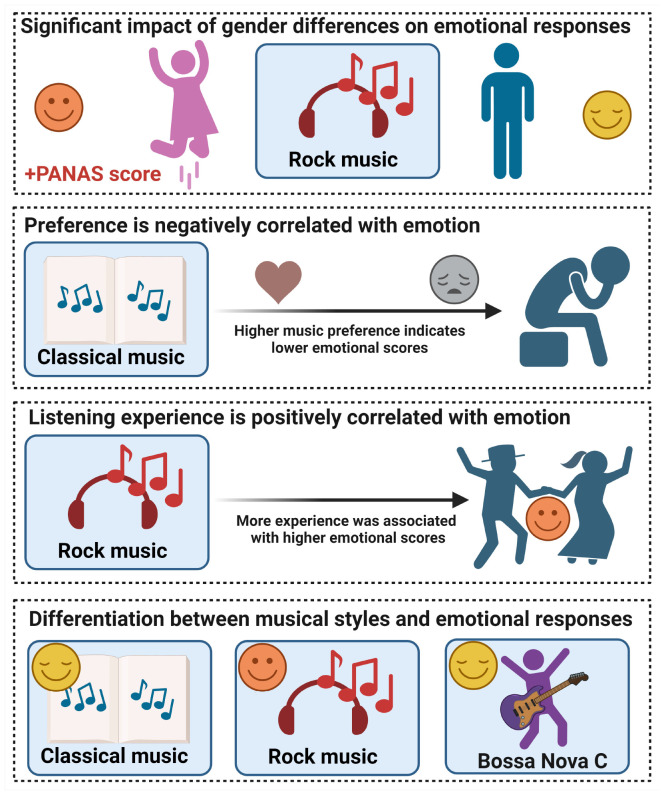
Differences in the effects of gender, music preference, and listening experience on adolescent emotional responses.

## Introduction

Music, as a universal cultural phenomenon, permeates all aspects of human life. Whether in daily entertainment, ceremonial celebrations, or professional artistic creation, music plays a pivotal role in the development of human societies (Malloch and Trevarthen, 2018). In recent years, the emotional effects of music have garnered increasing attention across various disciplines, including psychology, education, and medicine (Wang et al., 2024; Liu et al., 2018). Research shows that music not only influences individual emotions and psychological states but also serves as a potential intervention tool for improving mental health (Tang et al., 2020). For instance, music therapy has demonstrated significant benefits in managing emotional disorders such as anxiety and depression (Sun et al., 2021; Tang et al., 2020). For adolescents, who are in a critical stage of rapid psychological and physiological development, emotional responses to external stimuli are particularly sensitive (Iimura, 2021; Rapee et al., 2019). Thus, studying the impact of music on adolescent emotional responses not only helps to elucidate the mechanisms of music’s effects but also provides valuable insights for music education and psychological health interventions.

Music, as a potent emotional trigger, can significantly shape listeners’ subjective emotional experiences through its structural features such as rhythm, mode, melody, and harmony. Empirical studies have shown that fast-tempo music typically induces higher levels of pleasure and arousal, while slow-tempo music is more likely to evoke reflection and relaxation (Liu et al., 2018). Theoretically, the Basic Emotion Theory posits that emotions are evolutionarily adaptive and culturally universal. The Dimensional Model structures emotions along valence and arousal dimensions and is widely applied in music emotion research (Grekow, 2016). The Appraisal Theory further emphasizes that emotional responses are shaped not only by external stimuli but also by individual evaluations, cognitive processing, cultural context, and personal experience. Music can evoke complex emotional experiences through mechanisms such as resonance, memory activation, rhythmic entrainment, and contextual association. Therefore, studying the effects of musical arrangement on adolescents’ emotional responses requires an integrated theoretical framework that combines structural features of music with individual differences, grounded in Basic Emotion Theory, the Dimensional Model, and Appraisal Theory, to better understand the mechanisms of music-induced emotion.

In recent years, numerous studies have explored the mechanisms by which music influences emotional responses. Specific musical features, such as melody, rhythm, harmony, and arrangement, are thought to evoke distinct emotional experiences. For instance, upbeat rhythms are often associated with positive emotions, while slower melodies are more likely to induce reflective or melancholic moods. Additionally, existing research highlights that individual emotional responses to music may be influenced by various factors, including gender, cultural background, music preferences, and personal musical experiences). Musical preference refers to an individual’s subjective inclination toward specific music styles or genres. Listening experience denotes the frequency and breadth of unstructured musical exposure. Formal music training involves skill-based learning acquired in structured educational settings. However, there is a lack of systematic empirical research that integrates “musical arrangement” with these individual characteristic variables—especially within the context of adolescence.

To address the existing research gap, this study proposes the following core questions: Do adolescents exhibit significant differences in emotional responses when exposed to different musical arrangements (classical, rock, bossa nova)? Does gender moderate these emotional responses? Do musical preferences and listening experiences influence the intensity of emotional reactions? And does formal music training have a measurable effect? Based on these questions, several hypotheses are formulated: First, the rock and bossa nova versions are expected to elicit stronger emotional arousal than the classical original, potentially due to rhythm intensity and its activation of the brain’s reward system (Foster Vander Elst et al., 2021). Second, females are hypothesized to report higher emotional ratings across all versions, reflecting their greater emotional sensitivity (Bek et al., 2021). Third, musical preference is expected to show direction-specific negative correlations with emotional responses—for example, a stronger preference for rock may be negatively associated with emotional ratings for the rock version (B), and a stronger preference for pop may negatively correlate with ratings for the classical version (A). Furthermore, an unstructured music listening experience is predicted to have a positive influence on emotional ratings, whereas years of formal, structured music training are not expected to have a significant effect. This could be explained by a potential decoupling between technical skill acquisition and emotional processing (Pizzie et al., 2020).

This study aims to systematically examine adolescents’ positive emotional responses to different musical arrangements, while analyzing the moderating roles of gender, musical preference, and music-related experience. Through rigorous experimental design and validated emotion assessment tools, the study not only fills theoretical gaps in music psychology but also offers practical implications for music psychology. The findings may inform personalized music therapy approaches and provide theoretical support for adolescent psychological interventions and the design of music curricula.

## Materials and methods

### Participants and study design

This study employed a descriptive cross-sectional design, with data collected via a structured online questionnaire. A stratified random sampling strategy was used to ensure socioeconomic diversity. Based on regional gross domestic product (GDP) per capita, six public high schools were randomly selected from a government-issued list provided by the Ministry of Education of mainland China. The sample included three key high schools in provincial capitals, two regular high schools in prefecture-level cities, and one county-level high school. Music teachers distributed questionnaires in their respective classrooms through an online platform. Students participated voluntarily and provided informed consent electronically.

Sample size determination was guided by an a priori power analysis conducted using G*Power 3.1. A multiple linear regression model was specified with five predictors (gender, formal music training, music preference, listening experience, and age), a medium effect size (*f*^2^ = 0.15), a significance level of α = 0.05, and a statistical power of 0.80. The analysis yielded a minimum required sample size of 92. To account for potential invalid responses, a total of 150 students were recruited for the study.

Eligibility criteria included: (1) current enrollment in a regular high school, with normal hearing and vision, and no major psychological impairments; and (2) participation in music courses based on the 2022 revised national high school music curriculum for at least one academic year. The institutional ethics committee approved the study protocol. All procedures complied with ethical standards, including anonymous data collection, encrypted storage, and group-level reporting to ensure individual confidentiality.

Data cleaning followed a multi-step protocol. First, 30 responses were excluded because they failed attention-check items or contained logical inconsistencies. Next, univariate outliers were identified using boxplot analysis (1.5 times the interquartile range), supplemented by review of response patterns and completion times to detect careless or invalid responding. A final analytic sample of 120 valid questionnaires was retained for statistical analysis (Supplementary Figure S1). A detailed flowchart of the inclusion and exclusion process is provided in Supplementary Figure S2.

### Music stimuli

The music stimulus selected was *Canon in D Major* by Johann Pachelbel, adapted from the 2022 edition of the high school music textbook published by the People’s Music Publishing House. Based on this version, three distinct arrangements—labeled Versions A, B, and C—were developed for experimental use. The audio materials were derived from either the original composition or existing online adaptations (Table 1) and were encrypted and distributed via QR codes, which participants scanned for playback. Structurally, *Canon* in D comprises three staggered voices, each entering two measures apart, accompanied by a repeating ground bass that outlines an eight-chord harmonic progression. The piece integrates the strict contrapuntal form of a canon with the variation structure of a chaconne, resulting in subtle yet continuous transformations that are “both delightful and imperceptible.” Version B incorporated rock elements characterized by strong beats, fast tempos, and energetic melodic lines. This rhythmic intensity was designed to elicit heightened physiological arousal through strong metric accents and high-frequency spectral content, potentially activating amygdala-mediated arousal circuits. Version C integrated bossa nova elements, employing syncopated rhythms and suspended harmonic resolutions to enhance dopaminergic activity in the striatum, thereby evoking a sense of reward anticipation. Compared with Version A (classical), these arrangements introduced distinct auditory features in terms of rhythm, loudness, spectral centroid, and timbral parameters (see Supplementary Table S1), which were further supported by spectrograms, amplitude envelopes, and MFCC heatmaps (Supplementary Figures S3A–C). The audio structure and corresponding analysis scripts are provided in Supplementary material 1.

**TABLE 1:** Music analysis of three versions.

ID	Canon	Style	Composer	Genre	Instrument	Tempo (bpm)
A	Original Edition	Classical	Pachelbel	D major	Strings	♩ = 76
B	Arrangement 1	Rock	VIGI	D major	Electroacoustic band	♩ = 200
C	Arrangement 2	Bossa nova	Ko Yumi	D major	Piano	Freely

### Questionnaire design

**FIGURE 1:**
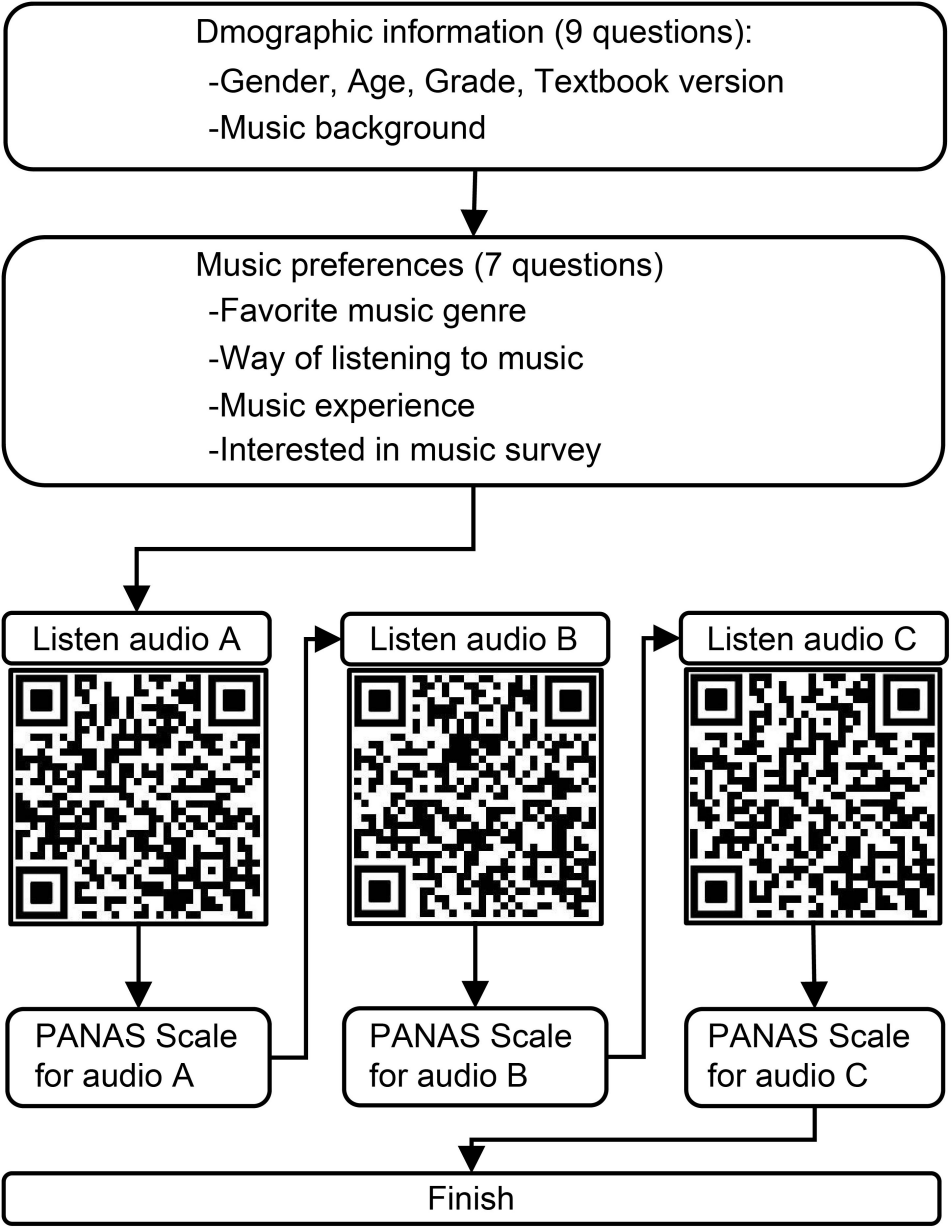
Flowchart of survey process on adolescents’ positive emotional responses to different music arrangements.

The questionnaire is divided into three sections (Supplementary material 2). The first section involves the collection of personal information and a survey of musical background, including nine questions about participants’ basic information, such as gender, age, grade, textbook version, and educational experience. The second section focuses on collecting music preferences and musical habits, comprising seven music-related questions, such as favorite music genre, listening techniques, and musical experience, to form the basis of the investigation into music preferences. The third section assesses emotional experiences using the PANAS (Supplementary material 3). Participants sequentially scanned each QR code (QR-A, B, and C) to listen to audio A, B, and C, and then rated the Positive Emotions Scale. Participants rested for 30 s between each task (Figure 1). Finally, they clicked to submit the questionnaire.

### PANAS

Participants rated each item based on their feelings after listening to the music. The rating scale ranged from 1 to 5, representing varying intensities from “not at all” to “very strongly.” Responses to each item were used to calculate overall positive and negative affect scores.

### Acquisition of electrodermal activity and heart rate variability

EDA was continuously recorded via Ag/AgCl finger electrodes at a sampling rate of 1,000 Hz. Mean skin conductance responses (SCRs, in μS) were extracted for each music segment using Ledalab software (v3.4.9), reflecting sympathetic arousal. HRV was measured using a Polar H10 chest strap sensor, with RMSSD computed from interbeat intervals during each listening period to reflect parasympathetic activity. All physiological recordings were synchronized with the onset of music using Presentation software (Neurobehavioral Systems) and processed offline. Participants remained seated and were instructed to minimize movement and avoid verbal communication throughout the session.

### Statistical analysis

All statistical analyses were performed using GraphPad Prism software (version 10.0). Descriptive statistics were used to summarize participant characteristics. Statistical significance was set at a two-tailed alpha level of 0.05. To assess the strength and direction of associations between continuous variables, Pearson’s correlation coefficient (*r*) was used for data meeting the assumption of bivariate normality, while Spearman’s rank correlation coefficient (ρ) was applied for non-normally distributed or ordinal variables. Group differences in continuous outcomes were examined using unpaired (independent) *t*-tests for normally distributed data. Multivariate linear regression analysis was conducted to identify predictors of emotional response scores. A *p* < 0.05 was considered statistically significant.

Assumption diagnostics were performed for all major analyses, including ANOVA, Pearson correlation analysis, and multiple linear regression, to ensure the validity of statistical inferences. The normality of main variables and regression residuals was assessed using the Shapiro-Wilk test and probability-probability (P-P) plots; homogeneity of variances across groups was evaluated with Levene’s test; multicollinearity in regression analyses was examined using the variance inflation factor (VIF); and independence of errors was assessed with the Durbin-Watson statistic. The diagnostic results indicated that all statistical assumptions were met (Supplementary Table S2; Supplementary Figure S4).

## Results

### Participant characteristics analysis

A total of 120 valid responses were included in the final analysis (Table 2). Of the participants, 51 (42.5%) were female and 69 (57.5%) were male. The majority were aged 16–17 years (*n* = 63, 52.5%), followed by 15–16 years (*n* = 42, 35.0%) and 17–18 years (*n* = 15, 12.5%). Most participants (*n* = 111, 92.5%) were in their first year of high school, with only 9 students (7.5%) in their second year. Regarding school-based music education, 27 participants (22.5%) used textbooks from the People’s Music Publishing House, while 93 (77.5%) used textbooks from the Education Publishing House. A total of 30 students (25.0%) reported receiving extracurricular music education, whereas 90 (75.0%) did not. Twelve participants (10.0%) identified as art-track students, and 108 (90.0%) as non-art-track students. Regarding interest in music, 39 participants (32.5%) reported a neutral attitude, 42 (35.0%) indicated they liked music, and 39 (32.5%) expressed strong liking. Pop music was the most preferred genre (*n* = 51, 42.5%), followed by traditional music (*n* = 24, 20.0%), classical music (*n* = 18, 15.0%), vocal music (*n* = 15, 12.5%), and instrumental music (*n* = 12, 10.0%).

**TABLE 2:** Demographic information of participants.

Parameters	*N* (%)
Responses	120 (100.0%)
**Gender**	
Female	51 (42.5%)
Male	69 (57.5%)
**Age (years)**	
15–16	42 (35%)
16–17	63 (52.5%)
17–18	15 (12.5%)
**Grade**	
Freshman	111 (92.5%)
Sophomore	9 (7.5%)
**Music textbook version used**	
People’s music publishing house	27 (22.5%)
People’s education publishing house	93 (77.7%)
**Music education out school**	
Have	30 (27.02%)
Haven’t	90 (72.98%)
**Art specialty student**	
Yes	12 (10%)
No	108 (90%)
**Love music**	
Neutral	39 (32.5%)
Like	42 (35%)
Really like	39 (32.5%)
**Music preference**	
Pop music	51 (42.5%)
Traditional music	24 (20%)
Vocal music	15 (12.5%)
Instrumental music	12 (10%)
Classical music	18 (15%)

### Impact of music styles on emotional responses

**FIGURE 2:**
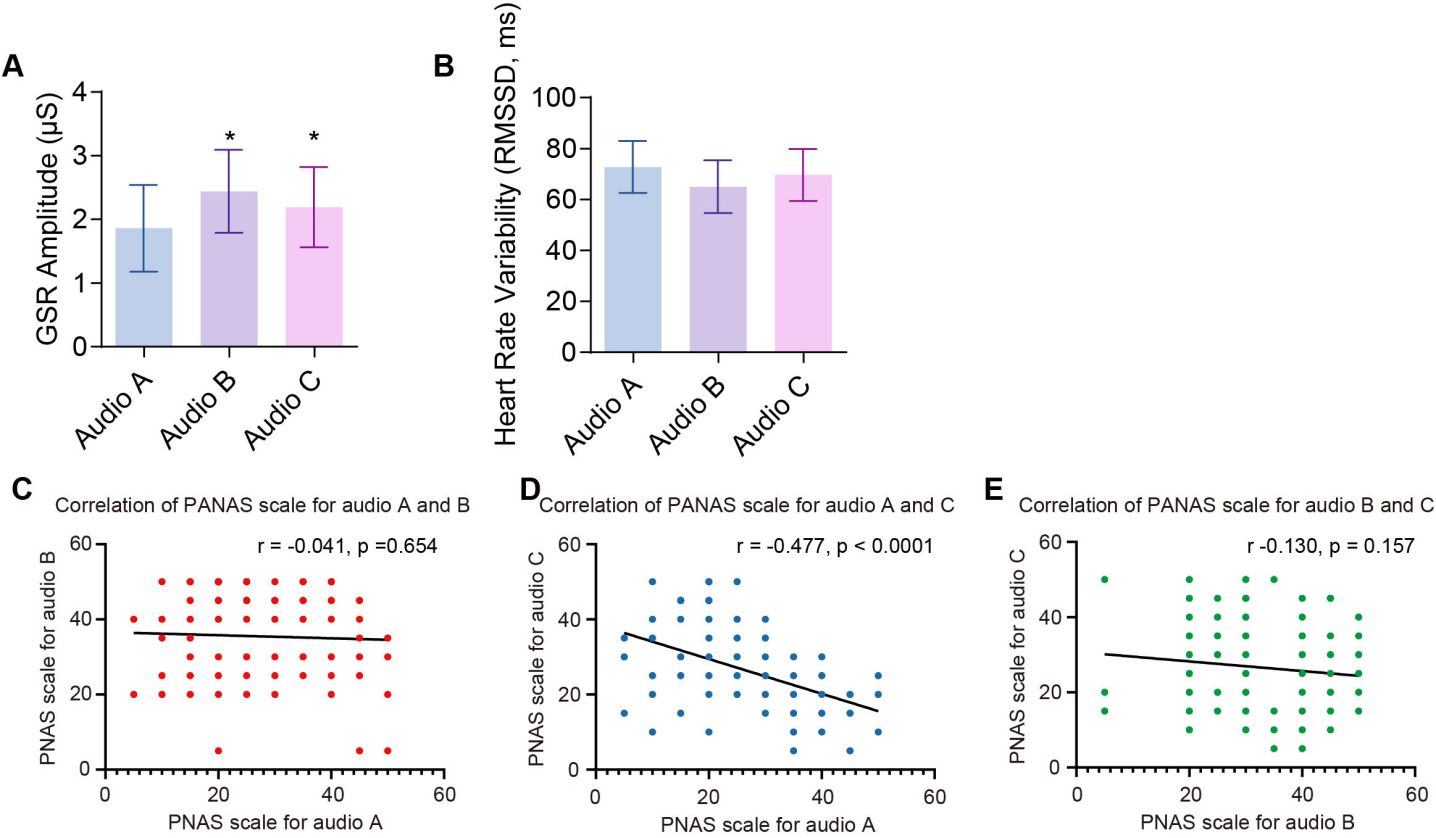
GSR, HRV, and PANAS score correlations across auditory stimuli of Audio A, B, and C. **(A)** Mean GSR amplitude for each audio condition. **(B)** Heart rate variability (RMSSD). **(C)** Correlation analysis of PANAS scores for Audio A and B. **(D)** Correlation analysis of PANAS scores for Audio A and C. **(E)** Correlation analysis of PANAS scores for Audio B and C.

The three musical styles exhibited a consistent pattern of differentiation in both subjective and physiological emotional indicators (Figure 2). Audio B elicited significantly higher GSR than the other versions, indicating a stronger emotional arousal effect. In contrast, Audio A showed the lowest GSR levels, but the HRV was higher, reflecting greater physiological regulatory capacity. Audio C demonstrated intermediate levels across all measures, suggesting a mild and balanced emotional response (Figures 2A,B). As shown in Figures 2C,D and Table 3, Pearson correlation analysis revealed a significant negative correlation between PANAS scores for Audio A and C (*r* = −0.477, *p* < 0.0001). However, no correlation was found between PANAS scores for Audio A and B (*r* = −0.041, *p* = 0.654) or between Audio B and C (*r* = −0.130, *p* = 0.157). In summary, classical music, rock music, and bossa nova each exhibit distinct emotional regulation functions, highlighting the unique roles of music styles in shaping adolescent emotional responses.

**TABLE 3:** Correlation analysis between the PANAS scores of Audio A, B, and C.

		PANAS score for Audio A	PANAS score for Audio B	PANAS score for Audio C
PANAS score for Audio A	*R*	1	−0.041	−0.477**
	*P*		0.654	< 0.0001
	*N*	120	120	120
PANAS score for Audio B	*R*	−0.041	1	−0.130
	*P*	0.654		0.157
	*N*	120	120	120
PANAS score for Audio C	*R*	−0.477**	−0.130	1
	*P*	< 0.0001	0.157	
	*N*	120	120	120

**p < 0.01.

### Analysis of factors influencing PANAS scores

**FIGURE 3:**
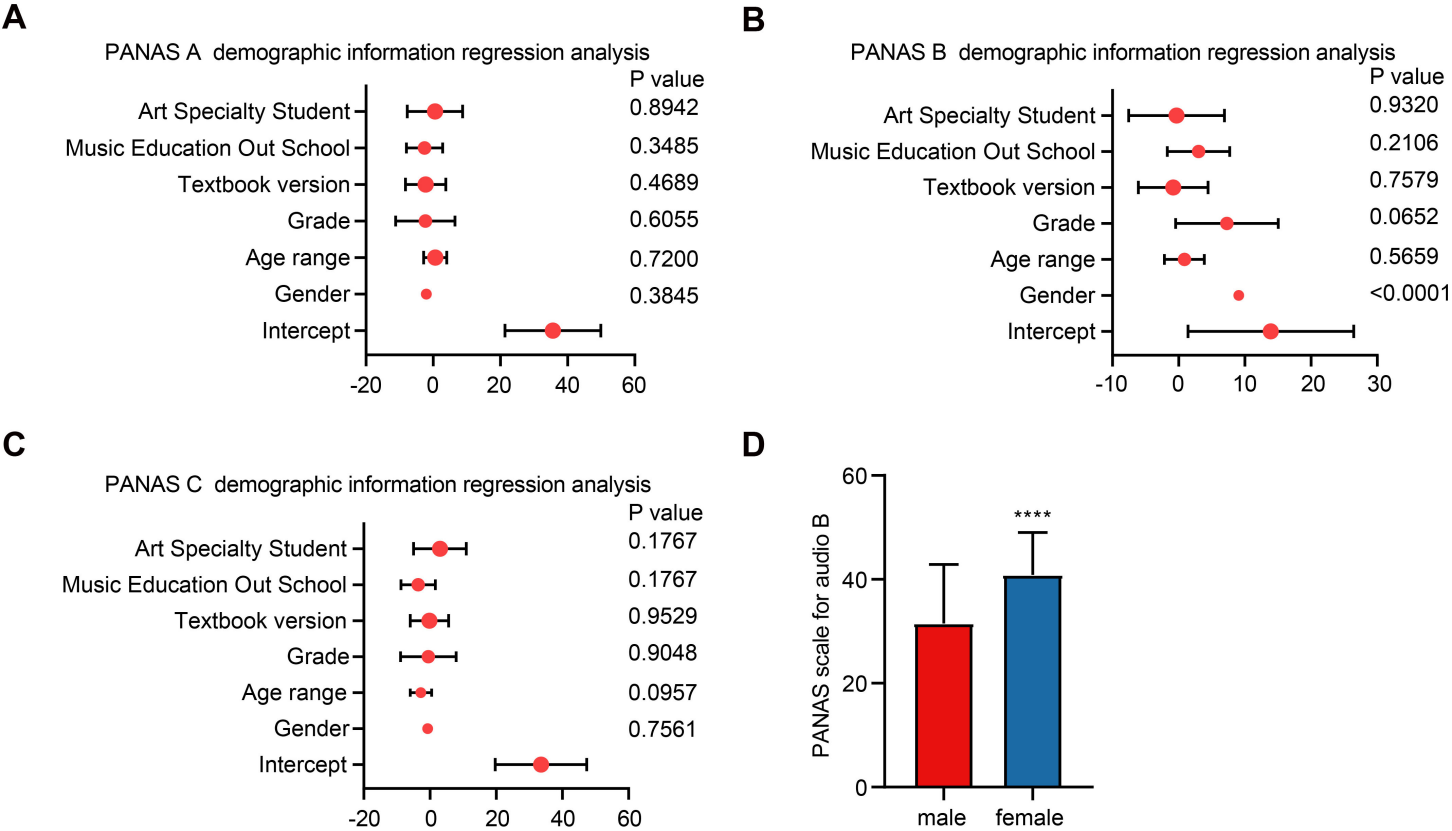
Demographic linear regression analysis on demographic factors of PANAS scores. **(A)** Multivariate linear regression analysis of factors influencing participants’ PANAS scores for Audio A. **(B)** Multivariate linear regression analysis of factors influencing participants’ PANAS scores for Audio B. **(C)** Multivariate linear regression analysis of factors influencing participants’ PANAS scores for Audio C. **(D)** Comparison of PANAS scores for Audio B between male and female participants.

Further analyzed whether factors such as gender, age, grade, music textbooks, and professional artistic training experience affected PANAS scores. Multivariate linear regression analysis revealed that gender (*p* < 0.001) significantly influenced participants’ PANAS scores for Audio B, while age, grade, music textbooks, professional artistic training experience, and whether participants were art specialists did not affect PANAS scores for Audio B. Additionally, none of these factors, including gender, age, grade, music textbooks, professional artistic training experience, or art specialization, influenced PANAS scores for Audio A or C (Figures 3A–C). An independent sample *t*-test further indicated a significant difference in PANAS scores for Audio B between males and females, with females scoring higher than males (Figure 3D).

### Effect of music preference on listening experience and PANAS scores

**FIGURE 4:**
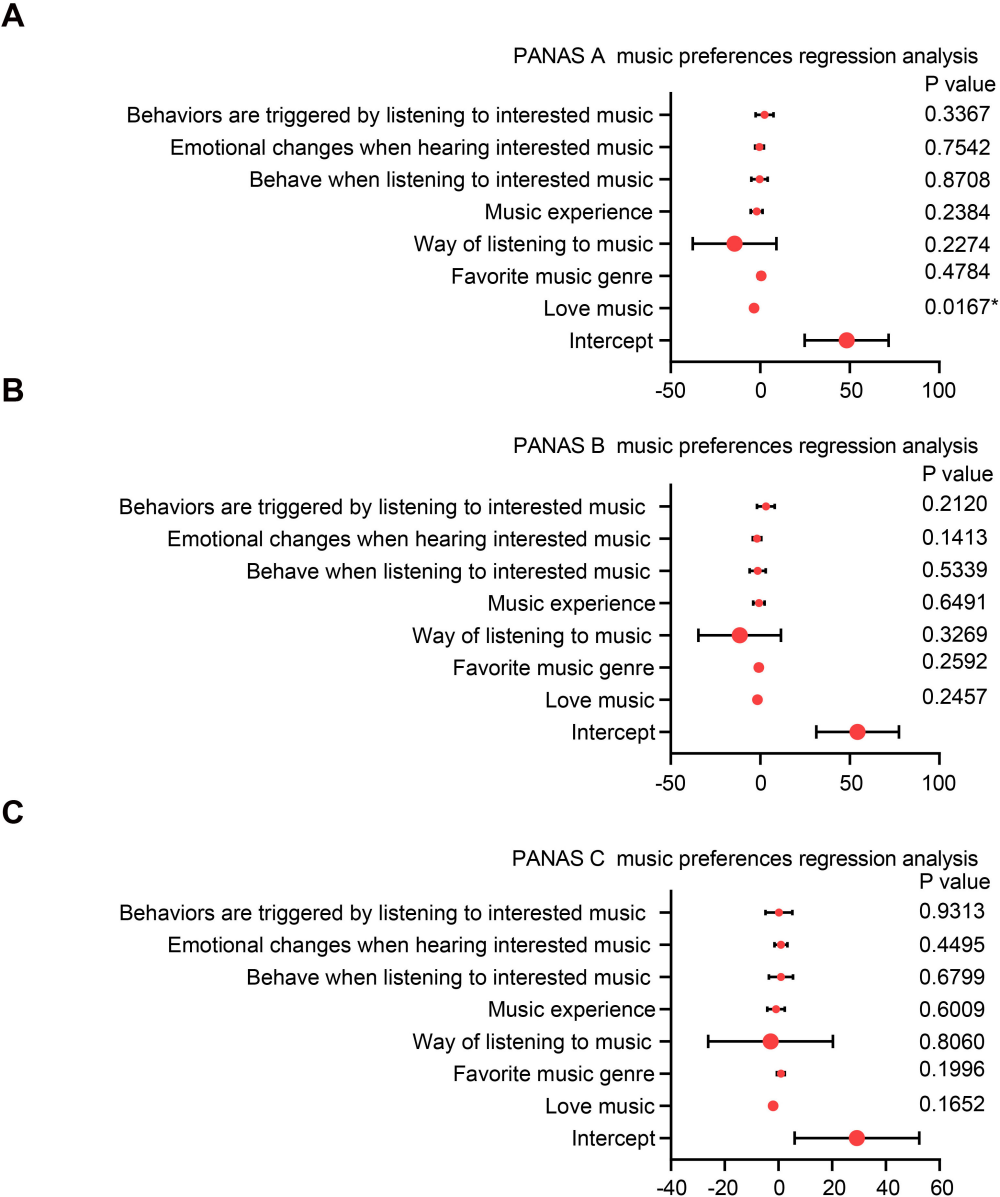
Demographic linear regression analysis on music preference factors of PANAS scores. **(A)** Multivariate linear regression analysis of music preference and habitual factors influencing participants’ PANAS scores for Audio A. **(B)** Multivariate linear regression analysis of music preference and habitual factors influencing participants’ PANAS scores for Audio B. **(C)** Multivariate linear regression analysis of music preference and habitual factors influencing participants’ PANAS scores for Audio C.

Pearson correlation analysis revealed no statistically significant associations between self-reported music preference levels and PANAS scores following exposure to Audio A, B, or C (Table 4). However, multiple linear regression analysis indicated that music preference significantly predicted PANAS scores for Audio A (*p* < 0.05), but not for Audio B or C (Figure 4). Further correlation analysis showed a significant negative correlation between music preference and PANAS scores for Audio A (*r* = −0.332, *p* = 0.049), while correlations with Audio B (*r* = 0.067, *p* = 0.475) and Audio C (*r* = −0.129, *p* = 0.161) were not statistically significant (Table 5). Other music-related variables, including listening habits, musical experience, emotional regulation strategies, and behavioral tendencies, did not significantly predict PANAS scores for any of the audio excerpts (all *p* > 0.05).

**TABLE 4:** The relationship between music preference and the feeling of listening to Audio A, B, C.

		Feelings with Audio A	Feelings with Audio B	Feelings with Audio C
Music preference	*r*	0.031	0.067	0.060
	*p*	0.735	0.465	0.508
	*N*	120	120	120

**TABLE 5:** The relationship between music preference and PANAS scores of listening to Audio A, B, C.

		PANAS score for Audio A	PANAS score for Audio B	PANAS score for Audio C
Music preference	R(95% CI)	−0.179[−0.184, −0.174]	−0.098[−0.084, −0.122]	−0.114[−0.110, −0.118]
	*P*	0.049*	0.286	0.2144
	*N*	120	120	120
	*R* ^2^	0.03	0.24	0.02
	*Adj.R^2^*	−0.01	0.22	−0.02

*p < 0.05.

### Effect of listening experience on PANAS scores for audio versions

Pearson correlation analysis revealed a significant positive correlation between the experience of listening to Audio B and its corresponding PANAS score (*r* = 0.245, *p* = 0.006). However, no correlation was found between the experience of listening to Audio A or C and their respective PANAS scores (*p* > 0.05) (Table 6).

**TABLE 6:** The relationship between the feeling of listening and PANAS scores of listening to Audio A, B, C.

		PANAS score for Audio A	PANAS score for Audio B	PANAS score for Audio C
The feeling of listening	*r*	−0.039	0.245	−0.008
	*p*	0.667	0.006^**^	0.926
	*N*	120	120	120

**p < 0.01.

## Discussion

This study, based on 120 adolescents, found that the emotional effects of three music arrangements were significantly moderated by gender, musical preference, and listening experience. Females reported higher positive affect scores in response to Version A, supporting the conclusion that they are more sensitive to emotional stimuli (Xu et al., 2021). The listening experience had a positive effect on emotional responses, while music education showed no significant influence. Notably, musical preference was negatively correlated with positive affect, particularly for Version A, challenging the “preference enhances pleasure” hypothesis. Each arrangement exhibited distinct stylistic characteristics: Version A enhanced a sense of safety, Version B boosted arousal, and Version C promoted relaxation. These results reveal interactive mechanisms between music structure and individual differences, expanding current research perspectives.

This study provides valuable insights and challenges existing research across three key dimensions: gender differences, musical preferences, and musical experiences. First, regarding gender, females exhibited significantly higher positive emotional responses to Version A compared to males, supporting prior empirical findings that suggest greater emotional sensitivity in females across recognition, expression, and neural responses (Fischer et al., 2018; Lausen and Schacht, 2018; Bek et al., 2021). Although some studies have found gender effects in musical emotion responses to be non-significant, this study observed a robust gender main effect even after controlling for musical structure variables, indicating that gender may serve as a critical moderator in musical emotional responses.

Second, musical preference did not generally correlate positively with emotional responses. A significant negative correlation was observed in response to Version A. This contradicts the widely held belief that preferred music enhances emotional pleasure (Egermann et al., 2015), suggesting that multiple mechanisms modulate the relationship between preference and emotion. The complexity, rhythmic stability, and restrained emotional expression of classical music may lead listeners to engage in deeper aesthetic processing and cognitive engagement, eliciting more nuanced and possibly ambivalent emotional experiences (Vuoskoski and Eerola, 2011; Tsetsos et al., 2010). Furthermore, high emotional expectations associated with preferred music may not always be fulfilled, leading to an “expectation discrepancy” that reduces subjective ratings. In contrast, Versions B and C, with more direct emotional expressions and pronounced rhythmic features, rely more on bottom-up acoustic activation than on preference-driven cognitive pathways. Thus, individual preference had limited impact on emotional responses to these styles (Juslin and Västfjäll, 2008).

Additionally, musical education did not significantly predict emotional responses, diverging from studies suggesting that musical training supports emotional processing (Limb and Braun, 2008). Possible reasons include wide variation in educational levels, lack of emotional content in training, or insufficient depth. In contrast, frequent music listening experience significantly enhanced positive affect, supporting theories of familiarity-driven emotional resonance. Among adolescents, whose musical preferences are still developing and influenced by peer culture, educational environment, and social labeling, the connection between musical style and emotion is still maturing. This further suggests that preference plays a more variable and possibly opposite role in complex musical styles, whereas it has a limited impact in rhythmically driven genres.

The study highlights the multifaceted moderating roles of gender, preference, and experience in musical emotion responses, underscoring the need for future research to explore interactions between individual variables and musical structures. Moreover, it calls for deeper investigation into the dynamic evolution of musical preference and emotional mechanisms across cultures and developmental stages.

The three musical arrangements (Version A, B, and C) exhibited significant differences in eliciting emotional responses among adolescents, attributable not only to their acoustic properties but also to differences in the neural pathways that activate emotions. Version A, characterized by a spectral centroid around 280 Hz and a canon-like polyphonic structure, enhances attention and emotional stability. Its aesthetic experience relies on ascending pathways involving musical expectation, symbolic association, and memory processing, leading to deeper aesthetic contemplation and complex emotional experiences (Juslin et al., 2013). In contrast, Version B features a rock rhythm with a high event density (142 events/min) and prominent 1.2 kHz high-frequency components. It primarily activates the brainstem reflex and striatal dopamine systems, rapidly inducing high-arousal emotional states such as excitement and pleasure (Chȩć et al., 2019). Version C utilizes approximately 35 ms syncopations and suspended harmonies to create rhythmic anticipation, thereby activating the nucleus accumbens reward system. This evokes peak emotional responses, such as “chills,” and enhances listeners’ sense of social connectedness—a reward mechanism recently validated in empirical research (Mori, 2022).

Building on these findings, we propose a three-dimensional interaction model, “Structural Acoustics-Individual Preference-Cultural Context,” to explain the nonlinear relationship between music style and emotional response. First, the “expectation discrepancy mechanism” suggests that unmet high emotional expectations for preferred music may result in emotional letdown and negative ratings, consistent with the observed negative correlation between preference and PANAS scores for Version A (Juslin et al., 2022). Second, music arousal pathways systematically differ: Version B relies primarily on bottom-up reflexive mechanisms, while Version A engages top-down cognitive construction pathways (Juslin et al., 2022; Tuomas, 2009). Third, cultural resistance mechanisms are especially prominent among adolescents, who often use music to construct identity and express anti-mainstream stances. Thus, they may show emotional detachment from music perceived as “textbook-like” or “frivolous” (Bauer et al., 2025). From a developmental psychology perspective, adolescents are in the early stages of forming their musical preference systems, which are heavily influenced by education and peer culture. This results in unstable or even inverse relationships between subjective preference and emotional response (Saarikallio et al., 2014). This study not only reveals the heterogeneous mechanisms through which different musical arrangements evoke emotions in adolescents but also introduces an interdisciplinary framework to account for these effects, enriching the current landscape of music psychology and developmental neuroscience. Despite these findings, several limitations should be acknowledged. First, the use of three arrangements based on a single song restricts the generalizability of results. Future studies should include a broader range of musical materials to enhance ecological validity. Second, to control for questionnaire length and reduce online experimental burden, this study used only the positive subscale of the PANAS. While this allowed for a focus on target emotions such as pleasure, vitality, and interest, it excluded negative or mixed emotional states and music-specific emotions, like “chills” or “nostalgia.” Future research should consider using comprehensive scales such as the full PANAS or GEMS, as well as continuous measurement methods to capture dynamic emotional fluctuations aligned with musical structure.

Another limitation concerns the analytical approach. While Pearson correlations identified basic patterns (e.g., the negative association between preference and PA scores), they did not reveal more complex interactions among variables. Future studies should employ more robust methods such as linear mixed-effects models, multivariate regression, or ANCOVA. Regarding the sample, 92.5% were first-year high school students, limiting insights into developmental variations across adolescence. For example, older students may exhibit enhanced emotion regulation capacity due to increased neuroplasticity, such as greater thickness in the right superior temporal gyrus—differences not captured in this study.

Future research should broaden the scope across cultural, age, and social dimensions to further explore adolescents’ emotional responses to different musical arrangements. To improve the representativeness of musical materials, we recommend applying the “MUSE” principle (Multicultural, Universal Structure, Socio-Emotional Tagging, Experimental Control) to construct a multidimensional music sampling model. Practically, this study lays a theoretical foundation for personalized music therapy, supporting the design of individualized interventions based on gender, preference, and neural pathway differences, and enhancing music’s role in adolescent emotional regulation and mental health support.

## Conclusion

This study demonstrates significant differences in adolescents’ emotional responses to various music arrangements, particularly influenced by gender, music preferences, and personal musical experience. Female participants exhibited stronger positive emotional responses to certain music arrangements, whereas music education and artistic training showed no significant effect on emotional responses. On the other hand, music preferences were negatively correlated with emotional responses, while the listening experience had a significant impact (Graphic abstract). Physiological measures further revealed distinct arousal and regulation patterns across music styles, supporting the subjective findings. These findings highlight the complexity and diversity of adolescents’ emotional reactions to music arrangements, emphasizing the critical role of gender and individual differences in music perception.

## Data Availability

The raw data supporting the conclusions of this article will be made available by the authors, without undue reservation.

## Supplementary material

Supplementary Figure S1 Inclusion and exclusion flowchart.

Supplementary Figure S2 Flowchart of participant inclusion and exclusion.

Supplementary Figure S3 Acoustic feature visualizations of canon in D in classical, rock, and bossa nova styles. **(A)** Spectrograms; **(B)** Amplitude Envelopes; **(C)** MFCC Heatmaps.

Supplementary Figure S4 Normal P-P plots of regression standardized residuals for PANAS Scores. **(A)** Audio A. **(B)** Audio B.
