# Supplementary material for: Adolescent emotional responses to different music arrangements
Source: Front Psychol. 2025 Nov 12;16:1583665. doi: 10.3389/fpsyg.2025.1583665 (PMC12659694; doi:10.3389/fpsyg.2025.1583665)
Supplement: Supplementary file 1 [file Supplementary_file_1.docx]

/Supplementary/

├── audio/

│ ├── classical.wav

│ ├── rock.wav

│ └── bossa.wav

├── analysis/

│ └── acoustic_analysis.py

├── figures/

│ ├── spectrograms.png

│ ├── envelopes.png

│ └── mfcc_heatmaps.png

import librosa

import numpy as np

import matplotlib.pyplot as plt

def analyze_audio(file_path):

y, sr = librosa.load(file_path)

bpm = librosa.beat.tempo(y, sr=sr)[0]

rms = np.mean(librosa.feature.rms(y=y))

centroid = np.mean(librosa.feature.spectral_centroid(y=y, sr=sr))

mfcc = np.mean(librosa.feature.mfcc(y=y, sr=sr, n_mfcc=13), axis=1)

return bpm, 20 * np.log10(rms), centroid, mfcc

for label, path in {

"Classical": "classical.wav",

"Rock": "rock.wav",

"BossaNova": "bossa.wav"

}.items():

bpm, loudness, centroid, mfcc = analyze_audio(path)

print(f"{label}: BPM={bpm:.1f}, RMS={loudness:.2f} dB, Centroid={centroid:.1f} Hz")

print("MFCCs:", np.round(mfcc, 1))
