# Supplementary material for: Adolescent emotional responses to different music arrangements
Source: Front Psychol. 2025 Nov 12;16:1583665. doi: 10.3389/fpsyg.2025.1583665 (PMC12659694; doi:10.3389/fpsyg.2025.1583665)
Supplement: Supplementary file 2 [file Supplementary_file_2.docx]

**Survey on Adolescents' Music Preferences and Emotional Responses**

This questionnaire aims to investigate adolescents' preferences for different music versions and their emotional responses. All data are strictly confidential and will be used solely for research purposes. The questionnaire consists of three parts. Please answer each question based on your actual situation.

**Part 1: Personal Information and Musical Background**

**Your Gender:**

1. Male
2. Female

**Your Age Group:**

1. 15 or below
2. 16
3. 17
4. 18
5. 19 or above

**Your Grade Level:**

1. First-year high school
2. Second-year high school
3. Third-year high school
4. Repeating grade

**Music Textbook Used in Your School:**

1. High School Music Textbook by People's Music Publishing House (2022 Revised Edition)
2. High School Music Textbook by Higher Education Press
3. High School Music Textbook by Jiangsu Phoenix Education Publishing House
4. High School Music Textbook by Hunan Fine Arts Publishing House
5. Others (please specify the textbook name)

**Have you received professional artistic training outside of school arts education?**

1. Yes
2. No

**Types of Artistic Training Received:**

1. Vocal
2. Instrumental
3. Music production
4. Broadcasting and hosting
5. Drama performance
6. Dance
7. Others

**Response options:** 0: Participated but unfamiliar; 1: Familiar with the training; -3: Have not participated

**7. Duration of Professional Extracurricular Artistic Education:**

1. -3: Have not received any training
2. 1: Attended only once
3. 2: Attended a few times but for a short duration
4. 3: Received systematic training
5. 4: Currently undergoing professional training
6. 5: Previously trained but no longer participating

**Are you an art specialist (intending to apply to an art college or currently undergoing training)?**

1. Yes
2. No

**Music-Related Activities Participated In:**

1. Choral activities (e.g., choir, a cappella)
2. Instrumental ensemble activities (e.g., band)
3. Music appreciation activities (e.g., concerts)
4. Music creation (e.g., arrangement, personal video recordings like Karaoke apps)
5. Others

**Response options:** 1: Participated**;** 0: Did not participate

**Part 2: Music Preferences and Listening Habits**

**Do you like music?**

1. Strongly dislike
2. Dislike
3. Neutral
4. Like
5. Strongly like

**Your Preferred Music Types:**

1. Chinese traditional music
2. Vocal music
3. Instrumental music
4. Classical music
5. Pop music
6. Others

**Response options:** 1: Like; 0: Do not like

**Platforms You Use to Listen to Music:**

1. Mobile apps (e.g., music apps, short-video apps)
2. TV music channels
3. Radio
4. Portable players (e.g., CD player, MP3)
5. Others

**Response options:** 1: Use the platform**;** 0: Do not use the platform

**Your Reaction to Liked Music Types:**

1. Sway to strong rhythms
2. Hum along to melodic music
3. Check or share lyrics of interest
4. Search for similar music or artists
5. Analyze musical elements of liked styles
6. Others

**Response options:** 1: Have this reaction**;** 0: Do not have this reaction

**How You Handle Liked Music:**

1. Play it on loop or add to playlists
2. Save and share with friends
3. Publish on public platforms
4. Use it for recording or creating videos
5. Others

**Response options:** 1: Have this behavior; 0: Do not have this behavior

**Emotional Changes When Listening to Music:**

1. Emotional synchronization and resonance
2. Feel stronger emotions while listening
3. Emotional relief while listening
4. Inner calmness while listening
5. Others
6. No change

**Response options:** 1: Have this reaction**;** 0: Do not have this reaction

**Further Reactions to Liked Music:**

1. Listen several times
2. Sing or learn to sing along
3. Analyze musical elements
4. Play the music with an instrument
5. Create new compositions based on it
6. Others

**Response options:** 1: Have this reaction**;** 0: Do not have this reaction

**Have you listened to the audio "Who Says My Hometown Isn't Good"?**

1. Yes
2. No

**In which setting did you listen to it?**

1. 1: Listened alone
2. 2: Listened during social activities
3. 3: Listened in class
4. -3: Have not listened

**Part 3: Positive and Negative Affect Schedule (PANAS) Emotional Experience Survey**

**Steps:** Please scan the QR codes below (QR-A, QR-B, QR-C) in sequence to listen to Audio A, B, and C. After each audio, complete the PANAS scale rating. Rest for 30 seconds after each audio.

**Your feelings while listening to Audio A:
Rating options:**

1. Very negative
2. Negative
3. Slightly negative
4. Neutral
5. Slightly positive
6. Very positive

**At what point did your emotions peak while listening to Audio A?**

1. Beginning
2. Middle
3. End
4. Throughout

**Rate aspects of Audio A based on your experience:**

1. Rhythm
2. Melody
3. Timbre
4. Style
5. Atmosphere
6. Others

**Response options:** -2: Not applicable or no opinion**;** 1: Very dissatisfied**;** 2: Dissatisfied**;** 3: Slightly dissatisfied**;** 4: Neutral**;** 5: Satisfied**;** 6: Very satisfied

**Your feelings while listening to Audio B:
Rating options:**

1. Very negative
2. Negative
3. Slightly negative
4. Neutral
5. Slightly positive
6. Very positive

**At what point did your emotions peak while listening to Audio B?**

1. Beginning
2. Middle
3. End
4. Throughout

**Rate aspects of Audio B based on your experience:**

1. Rhythm
2. Melody
3. Timbre
4. Style
5. Atmosphere
6. Others

**Response options:** -2: Not applicable or no opinion**;** 1: Very dissatisfied**;** 2: Dissatisfied**;** 3: Slightly dissatisfied**;** 4: Neutral**;** 5: Satisfied**;** 6: Very satisfied

**Your feelings while listening to Audio C:
Rating options:**

1. Very negative
2. Negative
3. Slightly negative
4. Neutral
5. Slightly positive
6. Very positive

**At what point did your emotions peak while listening to Audio C?**

1. Beginning
2. Middle
3. End
4. Throughout

**Rate aspects of Audio C based on your experience:**

1. Rhythm
2. Melody
3. Timbre
4. Style
5. Atmosphere
6. Others

**Response options:** -2: Not applicable or no opinion**;** 1: Very dissatisfied**;** 2: Dissatisfied**;** 3: Slightly dissatisfied**;** 4: Neutral**;** 5: Satisfied**;** 6: Very satisfied

**Please describe your mood after listening to the selected audio:**Please provide a detailed description of your emotional feelings and any additional thoughts.

**Please click "Submit" after completing all the questions. Thank you for your participation!**
