# Supplementary material for: Adolescent emotional responses to different music arrangements
Source: Front Psychol. 2025 Nov 12;16:1583665. doi: 10.3389/fpsyg.2025.1583665 (PMC12659694; doi:10.3389/fpsyg.2025.1583665)
Supplement: Supplementary file 3 [file Supplementary_file_3.docx]

**Positive and Negative Affect Schedule (PANAS)**

Welcome! Thank you for participating in this emotion assessment experiment. In the previous emotional program evaluation experiment, you gained basic insights into emotional judgment through facial expressions. Now, please assess your current emotional experience truthfully across 18 dimensions. Thank you for your cooperation!

**Instructions:** Each dimension has five levels of evaluation: "1" represents "very slight or none at all," "2" represents "slight," "3" represents "moderate," "4" represents "strong," and "5" represents "very strong." Please try to avoid selecting "3." Thank you for your cooperation.

1. **Please enter your gender:**
   Male / Female
2. **Active**
   Very slight or none at all - Very strong (Rating: 1, 2, 3, 4, 5)
3. **Alert**
   Very slight or none at all - Very strong (Rating: 1, 2, 3, 4, 5)
4. **Distressed**
   Very slight or none at all - Very strong (Rating: 1, 2, 3, 4, 5)
5. **Enthusiastic**
   Very slight or none at all - Very strong (Rating: 1, 2, 3, 4, 5)
6. **Proud**
   Very slight or none at all - Very strong (Rating: 1, 2, 3, 4, 5)
7. **Afraid**
   Very slight or none at all - Very strong (Rating: 1, 2, 3, 4, 5)
8. **Nervous**
   Very slight or none at all - Very strong (Rating: 1, 2, 3, 4, 5)
9. **Happy**
   Very slight or none at all - Very strong (Rating: 1, 2, 3, 4, 5)
10. **Excited**
    Very slight or none at all - Very strong (Rating: 1, 2, 3, 4, 5)
11. **Scared**
    Very slight or none at all - Very strong (Rating: 1, 2, 3, 4, 5)
12. **Guilty**
    Very slight or none at all - Very strong (Rating: 1, 2, 3, 4, 5)
13. **Exhilarated**
    Very slight or none at all - Very strong (Rating: 1, 2, 3, 4, 5)
14. **Joyful**
    Very slight or none at all - Very strong (Rating: 1, 2, 3, 4, 5)
15. **Irritable**
    Very slight or none at all - Very strong (Rating: 1, 2, 3, 4, 5)
16. **Jittery**
    Very slight or none at all - Very strong (Rating: 1, 2, 3, 4, 5)
17. **Energetic**
    Very slight or none at all - Very strong (Rating: 1, 2, 3, 4, 5)
18. **Grateful**
    Very slight or none at all - Very strong (Rating: 1, 2, 3, 4, 5)
19. **Panic-stricken**
    Very slight or none at all - Very strong (Rating: 1, 2, 3, 4, 5)

**PANAS Results Interpretation**

After completing the PANAS scale, the scores for each item are summed to derive the Positive Affect (PA) and Negative Affect (NA) scores. These scores lead to the following preliminary conclusions:

**Positive Affect (PA):**

- High Score (40+): The participant experiences strong positive emotions, characterized by active, excited, and proud feelings, indicating an overall positive emotional state.
- Medium Score (20–39): The participant experiences moderate positive emotions, occasionally encountering positive feelings, but not intensely.
- Low Score (below 19): The participant experiences low positive emotions, potentially lacking joy or vitality, with a more neutral or negative emotional state.

**Negative Affect (NA):**

- High Score (40+): The participant experiences significant negative emotions, such as nervousness, fear, or distress, potentially under high stress or negative emotional states. Emotional management is recommended.
- Medium Score (20–39): The participant experiences moderate negative emotions, occasionally encountering negative feelings, but not intensely.
- Low Score (below 19): The participant experiences low negative emotions, indicating a relatively stable emotional state, with minimal interference from negative feelings.
