# Supplementary material for: Adolescent emotional responses to different music arrangements
Source: Front Psychol. 2025 Nov 12;16:1583665. doi: 10.3389/fpsyg.2025.1583665 (PMC12659694; doi:10.3389/fpsyg.2025.1583665)
Supplement: Supplementary file 4 [file Table_1.docx]

# Table S1. Acoustic Features of Canon in D Across Three Musical Styles.

| **Acoustic Feature** | **Classical** | **Rock** | **Bossa Nova** |
| --- | --- | --- | --- |
| Tempo (BPM) | 72 | 112 | 94 |
| Average Loudness (RMS, dB) | -16.2 | -11.0 | -14.5 |
| Spectral Centroid (Hz) | 830 | 1800 | 1390 |
| Amplitude Envelope Peak (s) | 29.1 | 17.5 | 20.8 |
| MFCCs (Mean, 1–13) | [143, 49, 32, …] | [121, 64, 39, …] | [134, 52, 34, …] |
| Primary Instrumentation | Strings, Piano | Electric Guitar, Drums | Acoustic Guitar, Percussion |
| Genre Identification Accuracy | 90% | 95% | 85% |
