# Supplementary material for: Adolescent emotional responses to different music arrangements
Source: Front Psychol. 2025 Nov 12;16:1583665. doi: 10.3389/fpsyg.2025.1583665 (PMC12659694; doi:10.3389/fpsyg.2025.1583665)
Supplement: Supplementary file 5 [file Table_2.docx]

# Table S2. Statistical Assumption Diagnostics for Main Analyses.

| Assumption | Test / Indicator | Variable / Model | Value / Statistic | p-value / Conclusion |
| --- | --- | --- | --- | --- |
| Normality | Shapiro-Wilk, P-P plot | PANAS, residuals | W = 0.98–0.99 | p > 0.05 (normality met) |
| Homogeneity of variance | Levene’s test | PANAS (grouped) | F = 1.21–2.05 | p > 0.05 (homoscedasticity met) |
| Multicollinearity | VIF | Predictors | 1.00–1.10 | VIF < 2 (no collinearity) |
| Independence of errors | Durbin-Watson | Residuals | 1.17, 1.60 | ~2 (no autocorrelation) |

Note: PANAS: Positive and Negative Affect Schedule; P–P plot: Probability–Probability plot; VIF: Variance Inflation Factor.
