# Supplementary figures and images for: Adolescent emotional responses to different music arrangements
Source: Front Psychol. 2025 Nov 12;16:1583665. doi: 10.3389/fpsyg.2025.1583665 (PMC12659694; doi:10.3389/fpsyg.2025.1583665)

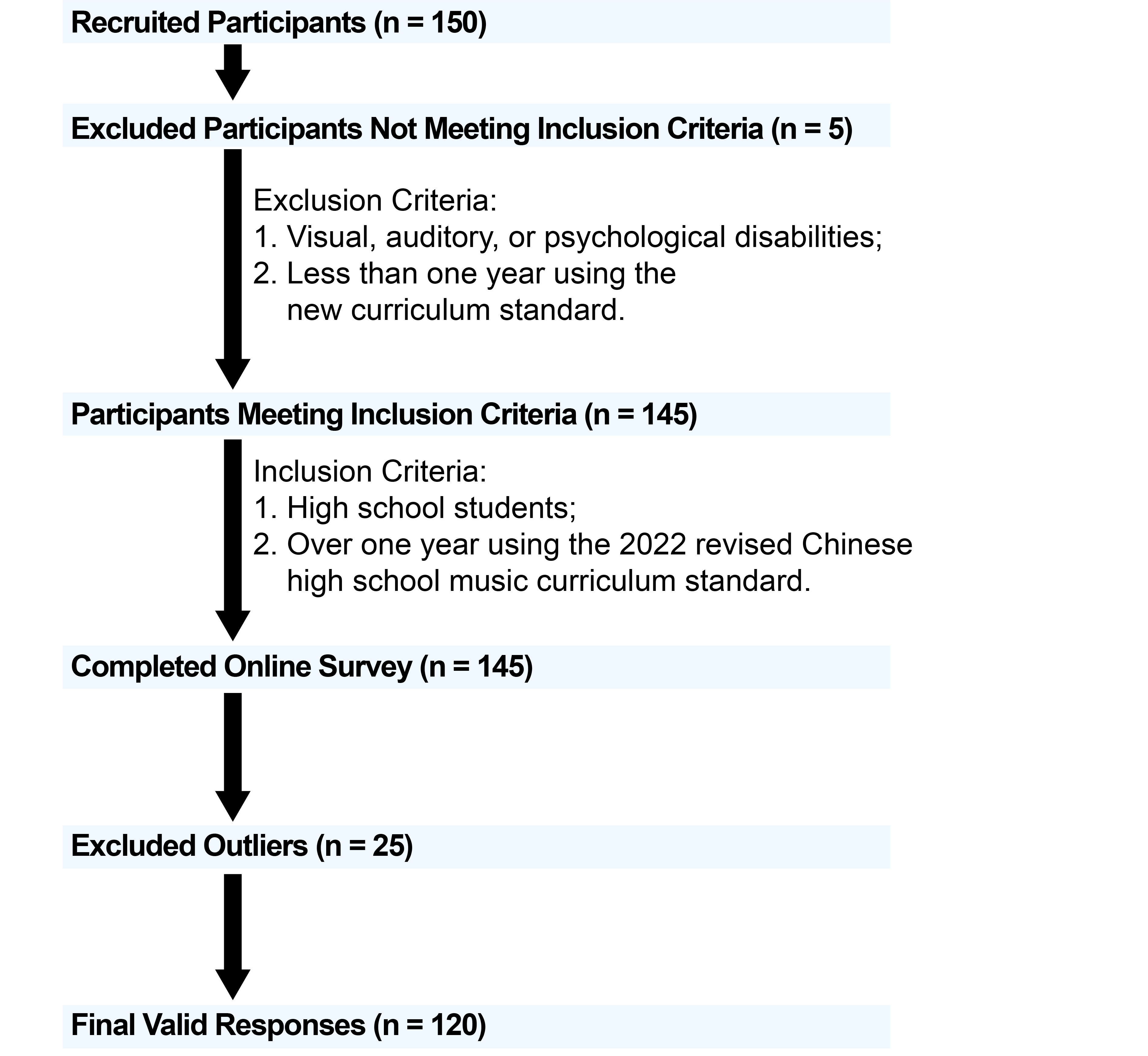

Supplement: Supplementary Figure S1 — Inclusion and exclusion flowchart. [file Image_1.jpeg]

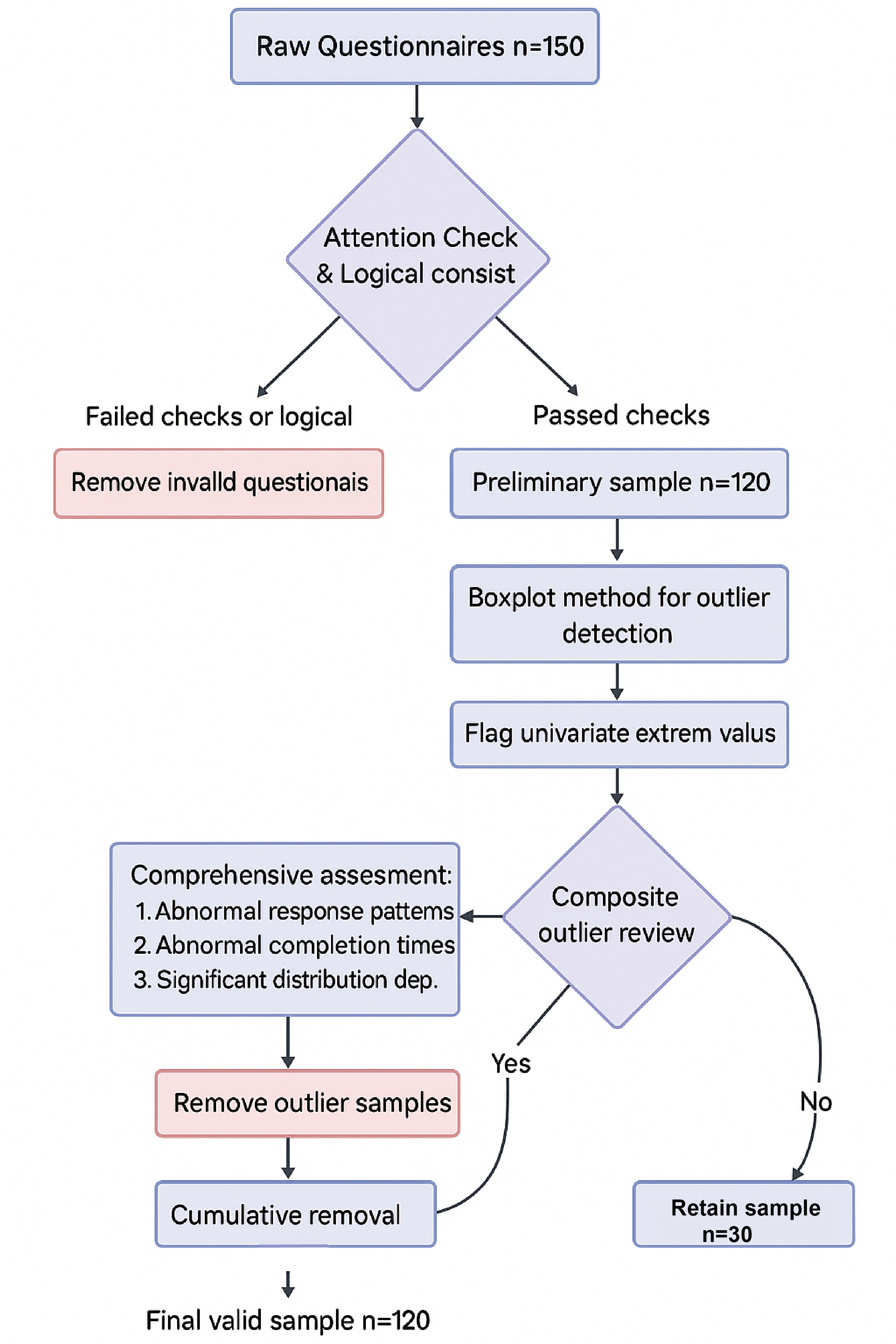

Supplement: Supplementary Figure S2 — Flowchart of participant inclusion and exclusion. [file Image_2.jpeg]

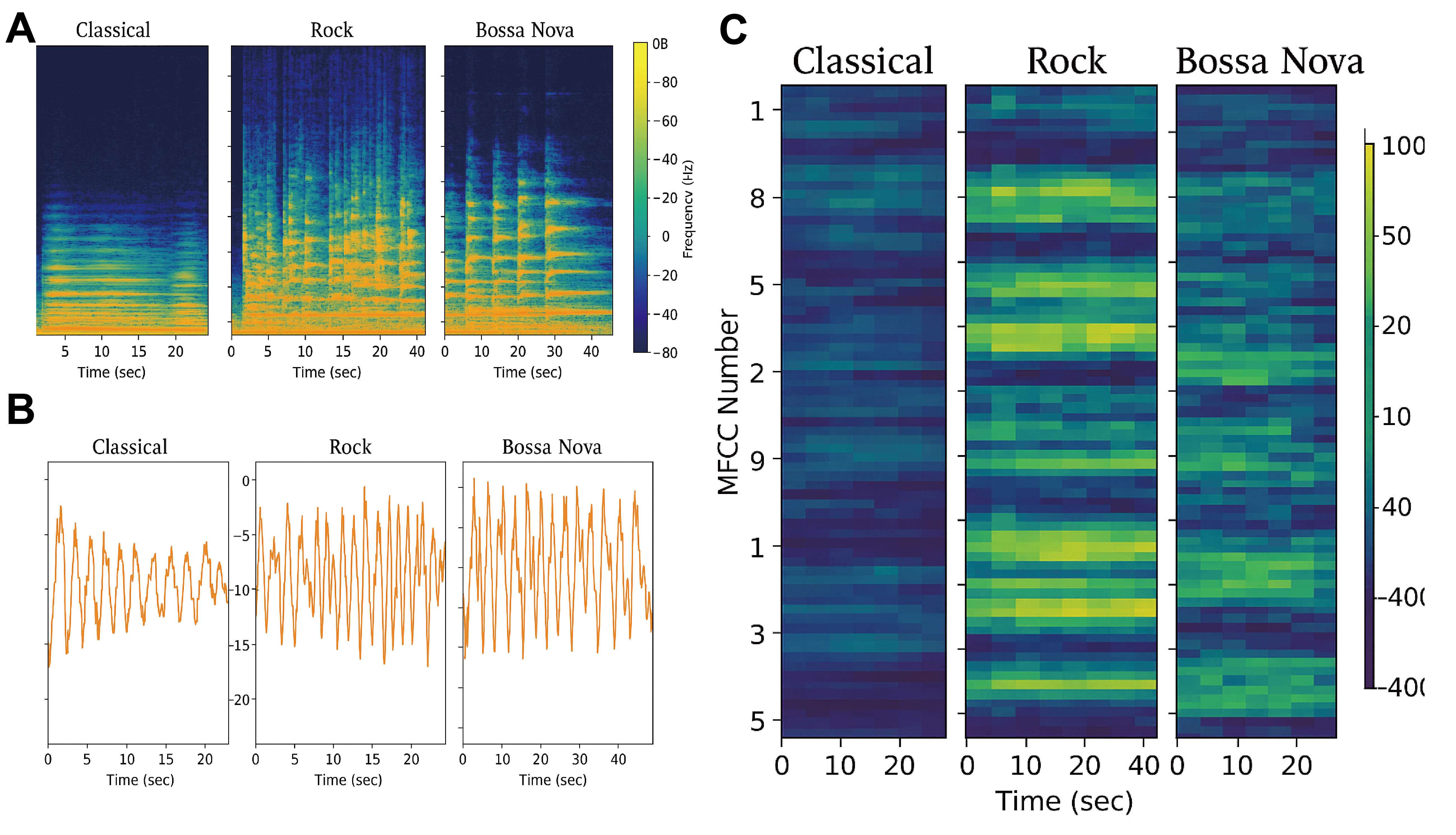

Supplement: Supplementary Figure S3 — Acoustic feature visualizations of canon in D in classical, rock, and bossa nova styles. (A) Spectrograms; (B) Amplitude Envelopes; (C) MFCC Heatmaps. [file Image_3.jpeg]

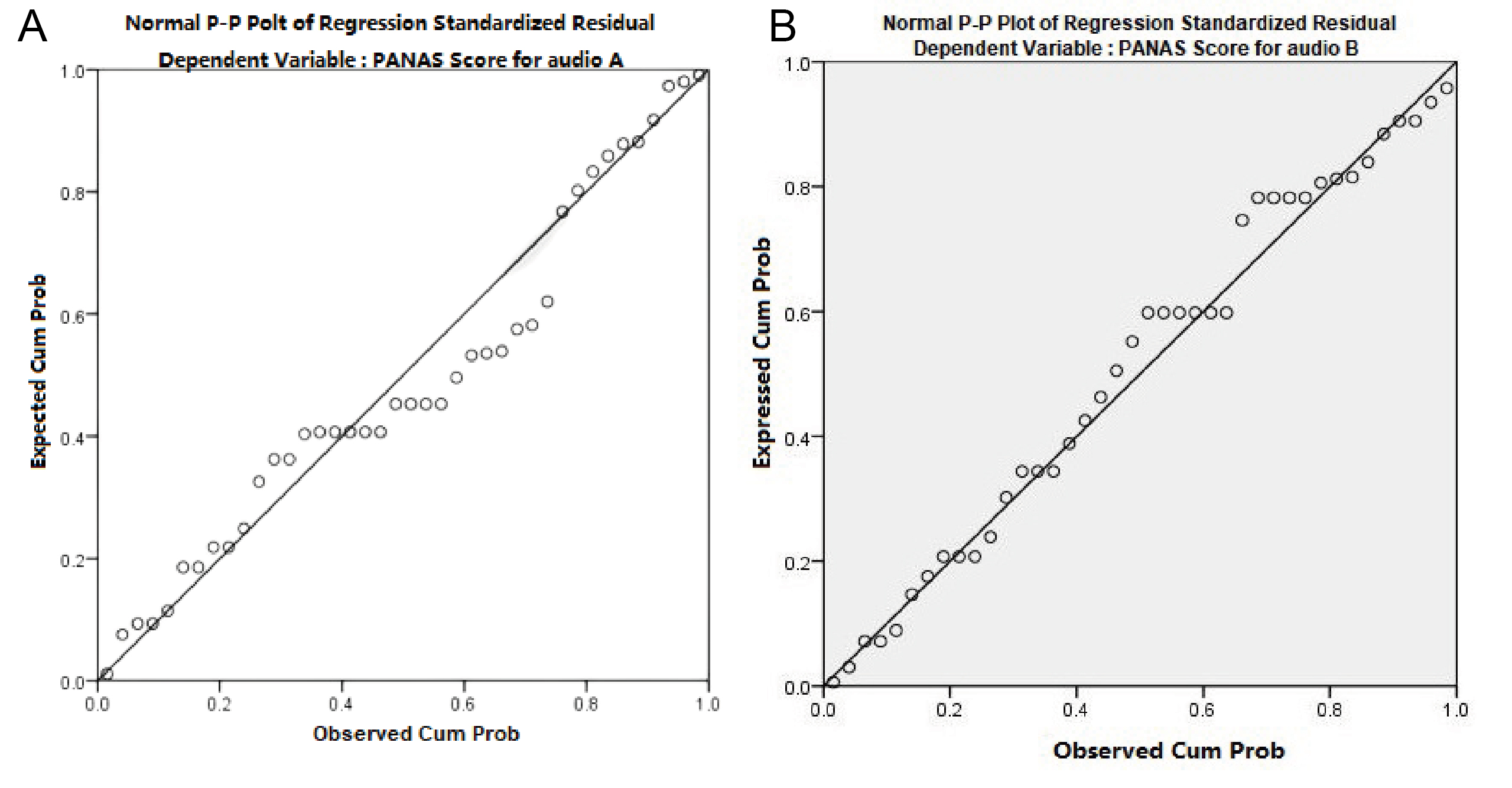

Supplement: Supplementary Figure S4 — Normal P-P plots of regression standardized residuals for PANAS Scores. (A) Audio A. (B) Audio B. [file Image_4.jpeg]
